# Exploring the Perspectives of Older Adults on a Digital Brain Health Platform Using Natural Language Processing: Cohort Study

**DOI:** 10.2196/60453

**Published:** 2024-11-18

**Authors:** Huitong Ding, Katherine Gifford, Ludy C Shih, Kristi Ho, Salman Rahman, Akwaugo Igwe, Spencer Low, Zachary Popp, Edward Searls, Zexu Li, Sanskruti Madan, Alexa Burk, Phillip H Hwang, Ileana De Anda-Duran, Vijaya B Kolachalama, Rhoda Au, Honghuang Lin

**Affiliations:** 1 Department of Anatomy and Neurobiology Avedisian School of Medicine Boston University Chobanian Boston, MA United States; 2 The Framingham Heart Study Avedisian School of Medicine Boston University Chobanian Boston, MA United States; 3 Beth Israel Deaconess Medical Center Harvard Medical School Boston, MA United States; 4 Department of Epidemiology Boston University School of Public Health Boston, MA United States; 5 School of Public Health and Tropical Medicine Tulane University New Orleans, LA United States; 6 Department of Computer Science Boston University Boston, MA United States; 7 Department of Medicine Avedisian School of Medicine Boston University Chobanian Boston, MA United States; 8 Slone Epidemiology Center Avedisian School of Medicine Boston University Chobanian Boston, MA United States; 9 Departments of Neurology Avedisian School of Medicine Boston University Chobanian Boston, MA United States; 10 Department of Medicine University of Massachusetts Chan Medical School Worcester, MA United States

**Keywords:** digital brain health, older adults, perspectives, semistructured interviews, natural language processing, mobile phone

## Abstract

**Background:**

Although digital technology represents a growing field aiming to revolutionize early Alzheimer disease risk prediction and monitoring, the perspectives of older adults on an integrated digital brain health platform have not been investigated.

**Objective:**

This study aims to understand the perspectives of older adults on a digital brain health platform by conducting semistructured interviews and analyzing their transcriptions by natural language processing.

**Methods:**

The study included 28 participants from the Boston University Alzheimer’s Disease Research Center, all of whom engaged with a digital brain health platform over an initial assessment period of 14 days. Semistructured interviews were conducted to collect data on participants’ experiences with the digital brain health platform. The transcripts generated from these interviews were analyzed using natural language processing techniques. The frequency of positive and negative terms was evaluated through word count analysis. A sentiment analysis was used to measure the emotional tone and subjective perceptions of the participants toward the digital platform.

**Results:**

Word count analysis revealed a generally positive sentiment toward the digital platform, with “like,” “well,” and “good” being the most frequently mentioned positive terms. However, terms such as “problem” and “hard” indicated certain challenges faced by participants. Sentiment analysis showed a slightly positive attitude with a median polarity score of 0.13 (IQR 0.08-0.15), ranging from –1 (completely negative) to 1 (completely positive), and a median subjectivity score of 0.51 (IQR 0.47-0.53), ranging from 0 (completely objective) to 1 (completely subjective). These results suggested an overall positive attitude among the study cohort.

**Conclusions:**

The study highlights the importance of understanding older adults’ attitudes toward digital health platforms amid the comprehensive evolution of the digitalization era. Future research should focus on refining digital solutions to meet the specific needs of older adults, fostering a more personalized approach to brain health.

## Introduction

Alzheimer disease (AD), a neurodegenerative disorder, currently has limited effective treatments, making early detection critically important for managing and preventing its progression. Recognizing this, many studies have identified biomarkers that can reveal the onset of the disease at an early stage [1,2]. These biomarkers, which range from blood markers and cerebrospinal fluid to brain imaging, offer promising ways for detecting AD before meaningful cognitive decline is evident [3-7]. However, these biomarkers often present challenges for continuous monitoring. A common disadvantage of these biomarkers—blood tests, cerebrospinal fluid analysis, and brain imaging—is the necessity for in-person visits. This poses a significant challenge for older adults who may face obstacles such as high costs, lack of or limited transportation options, and physical limitations, preventing them from traveling to health care facilities. With the advancement of digital technology, it has now become possible to collect a wide variety of data modalities remotely and in the convenience of where the person resides. Recently, numerous studies have begun to identify digital indices for early diagnosis of AD, such as voice [8-10], sleep patterns [11,12], and physical activity [13]. However, much of this research tends to look at each of the indicators in isolation. Integration of these diverse modalities is crucial to achieve a detailed and comprehensive characterization of the clinical profile. This approach not only enhances our understanding of the disease’s early signs more holistically but also paves the way for more personalized and proactive health care strategies.

However, deploying digital technology among older adults presents a unique set of challenges, such as usability concerns [14]. Our previous research has successfully implemented a participant-driven digital brain health platform and pioneered a multimodal data collection platform [15]. To determine scalability and sustainability, gaining a deeper understanding of older adults’ attitudes toward cognitive health monitoring devices is crucial. However, current research on older adults’ attitudes toward using a variety of different cognition-related technologies remains scarce. Most studies on the technology-related attitudes of older adults often investigate their viewpoints on broad technologies such as the use of the internet [16-18] and are unrelated to their views on monitoring cognitively related information. This gap highlights the need for further investigation into how older adults perceive and interact with digital health technologies, emphasizing the importance of designing accessible, user-friendly, and relevant digital health solutions that meet the specific needs and concerns about cognition and other related behaviors or functions within an aging population.

Semistructured interviews play a pivotal role in capturing the perspectives of older adults on technology [19]. By combining a predefined set of open-ended questions with the flexibility to explore topics in depth, these interviews offer a structured yet adaptable framework for understanding the unique experiences and attitudes of older adults. The efficacy of these interviews relies on the ability to extract crucial information effectively. Although qualitative data analysis methods evaluate layers of individuals’ thoughts, emotions, and personal narratives, they can be laborious and time-intensive, with inherent susceptibility to interpretive bias [20]. To address these challenges, natural language processing (NLP), with its foundations in computational linguistics, uses advanced algorithms to parse and analyze extensive text data systematically. This approach not only reduces the need for manual analysis but also offers a quantifiable measure of participants’ attitudes, thereby enhancing the objectivity and efficiency of capturing the sentiment of older adults toward technology [21].

This study conducted interviews with older adults from Boston University Alzheimer Disease Research Center (BU ADRC) who enrolled in the digital brain health program and used NLP to analyze their attitudes toward this multimodal data collection platform. The objective was to understand how older adults perceive and engage with these digital health technologies, with a focus on improving user experience, and the overall effectiveness of digital health solutions tailored to the older adult population.

## Methods

### Study Population

This study recruited participants from the Clinical Core of the BU ADRC. As one of the roughly 34 centers funded by the National Institute on Aging, the BU ADRC shares its data through the National Alzheimer Coordinating Center, enhancing collaborative studies on AD. A description of the BU ADRC’s scope and activities is detailed in a previous publication [15]. In 2021, we introduced to BU ADRC participants a digital platform for brain health, incorporating multiple digital data collection tools, including 9 remote technologies and 3 staff-guided digital assessments, to collect brain health–related measures [19]. The participants were offered a selection of technologies ranging from smartphone apps to wearable devices, with clear definitions of the commitment required for each technology [15]. The technology demonstration was tailored to participant preferences and conducted either remotely through videoconference or directly in person. During in-person visits, devices were set up and provided to the participants, while for remote sessions or subsequent quarterly follow-ups, the devices were prepared at the study center and mailed out. With a participant-driven approach, individuals selected their preferred technologies and were given a 14-day period to engage with these tools. The report of this study adheres to the STROBE (Strengthening the Reporting of Observational Studies in Epidemiology) statement (checklist presented in Multimedia Appendix 1) [22].

### Semistructured Interview

From April 2022 to July 2023, we carried out semistructured interviews following the participants’ initial 14-day assessment period with the platform to gain insights into their perceptions of the digital brain health platform. These interviews were designed to delve into specific areas: (1) to explore the experiences and opinions of the users regarding the platform; (2) to investigate any behavioral or health changes prompted by using the technology; (3) to assess the likelihood of sustained usage; and (4) to understand any health-related factors that could influence the adoption of the technology. The duration of these conversations typically ranged from 15 to 30 minutes. Conducted virtually through Zoom (Zoom Video Communications), the interviews were digitally recorded and transcribed verbatim using Datagain (Datagain Transcription Services). This tool has been widely used in health-related studies due to its adherence to HIPAA (Health Insurance Portability and Accountability Act) standards [23-25]. We further reviewed the transcripts for accuracy and removed personally identifiable information.

### Text Preprocessing

Our text analysis began with a cleaning and standardization process aimed at discarding extraneous information and achieving consistency across all participant responses. Initially, we distinguished between the contributions of the speakers, explicitly omitting any data from the interviewers to focus solely on the inputs of the participants. The first step in this refinement process involved removing stop words—commonly occurring yet semantically sparse words in the English language such as “the,” “is,” and “and”—using NLTK [26]. These words, despite their prevalence, did not offer much meaningful content for analysis. Then, we proceeded to tokenize the text, breaking it down into individual words. This involved transforming all characters to lowercase to achieve consistency. In addition, we filtered out nonalphanumeric tokens to further purify the data set. Lemmatization was used to significantly reduce the variety of word forms, including different tenses such as the past tense, by converting words into their base or dictionary forms. The culmination of this preprocessing phase was the reconstruction of the refined words into coherent strings, which were then assembled into a list of processed texts.

### Word Count Analysis

We computed the median and IQR for sentence and word counts to capture the breadth and depth of the verbal contributions of the participants. Then, we delved deeper into the emotional undercurrents of the text by quantifying the frequency of positive and negative words and calculating the number of participants who mentioned each word. To facilitate this analysis, we used Bing Liu’s opinion lexicon, a widely used tool to mine and summarize the customer reviews of a product that categorizes words into positive and negative lists [27]. In addition, we graphically represented the 10 most frequent positive and negative words through word clouds, illustrating the prominence of specific sentiments within the interviews of the participants.

### Sentiment Analysis

A lexicon-based approach was used to conduct sentiment analysis at both the sentence and participant levels, using the sentiment scores as determined by the internal lexicon of TextBlob [28]. This approach allowed us to assign quantitative sentiment scores to the text, with polarity scores ranging from –1 (completely negative) to 1 (completely positive) and subjectivity scores spanning from 0 (completely objective) to 1 (completely subjective) [29]. These scores were used to assess the emotional tone and objectivity or subjectivity of the responses of the participants. The median and IQR for both polarity and subjectivity were calculated.

### Ethical Considerations

The procedures and protocols of the study were approved by the Institutional Review Board at the Boston University Medical Campus (H 405‐42). Written informed consent was obtained from all participants. The participants did not receive compensation for their involvement. For analysis purposes, the participants were assigned unique identification numbers to ensure anonymity.

## Results

### Cohort Description

Our study included 28 participants from the BU ADRC (mean age 70, SD 8 years; 15/28, 55.2% women; and an average of 17, SD 2 years of education).

### Word Count and Sentiment Analysis

**Figure 1:**
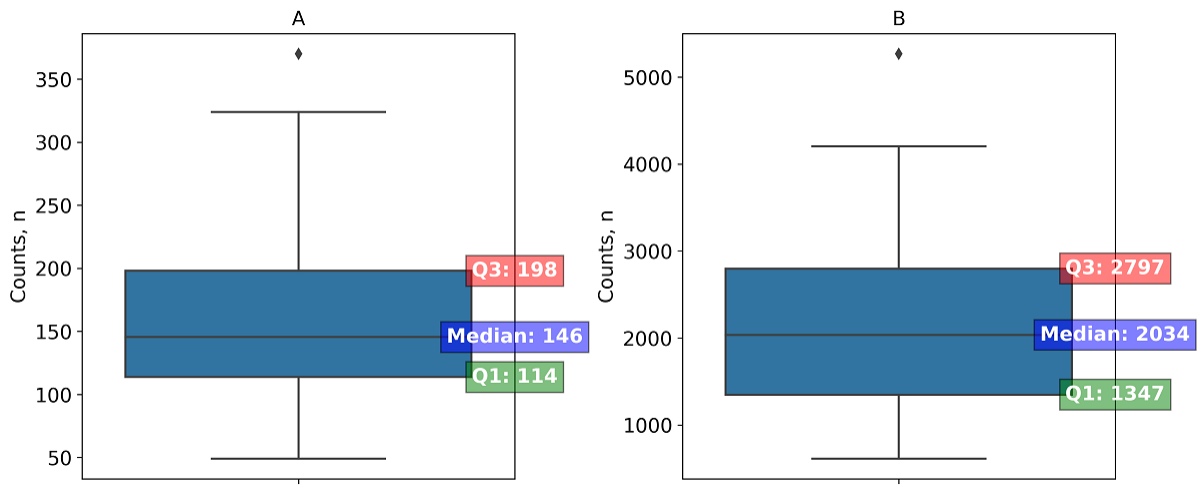
Descriptive statistics of textual features for participants identified during semistructured interviews: (A) sentence count and (B) word count.

**Figure 2:**
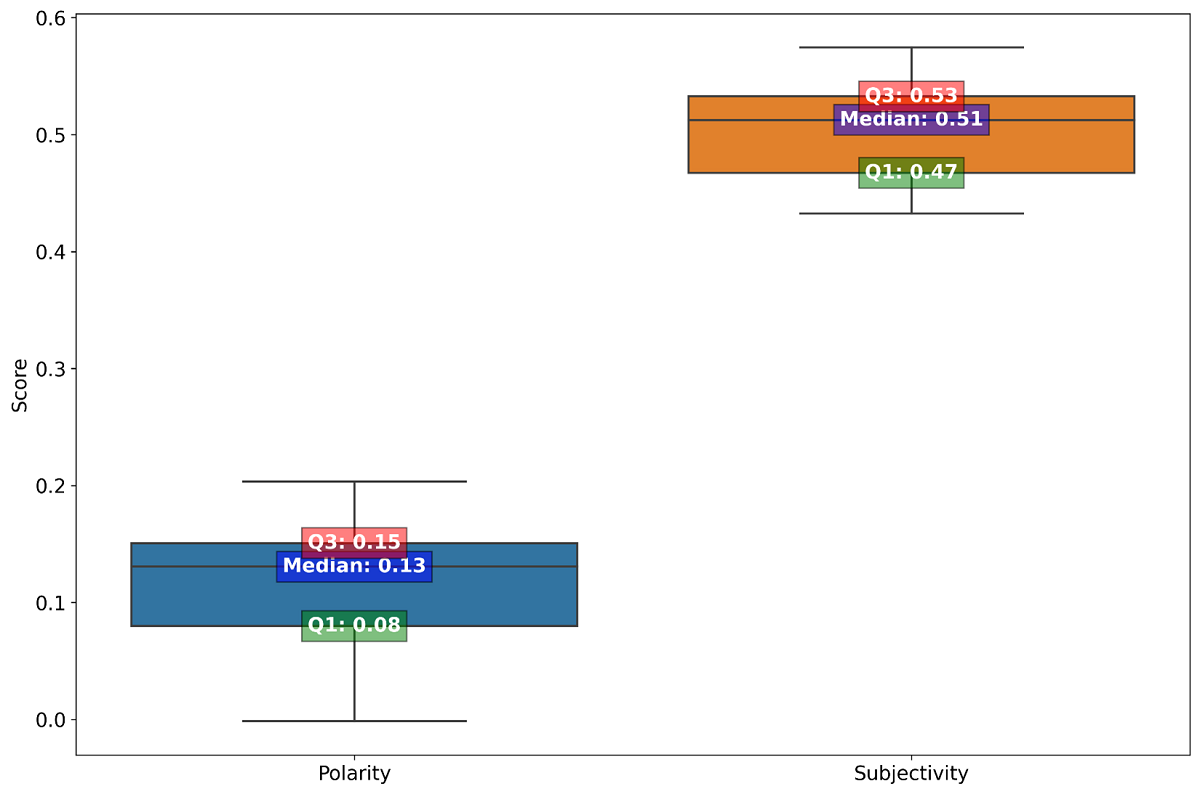
Descriptive statistics of polarity and subjectivity for participants identified during semistructured interviews.

Figure 1 provides a statistical summary of textual features and sentiment analysis for study participants. On average, each participant spoke 162 sentences, which included 2203 words. Table 1 summarizes the top 10 most used positive and negative terms by participants in the study. For positive terms, the word “like” is the most prevalent with a count of 540 mentions by all 28 participants. This is followed by “well” with 207 mentions and “good” with 152 mentions, both also widely used among participants. The list continues with other generally affirmative words such as “right,” “pretty,” and “better,” each mentioned by over 20 participants. On the negative side, “hard” is the term with the highest frequency, mentioned 38 times by 17 participants. The word “problem” follows closely with 37 mentions by 19 participants. Other negative terms like “difficult,” “uncomfortable,” and “frustrating” are also noted, each used by 10-12 participants. The terms “easy” and “work” close the list of positive terms, while “uncomfortable,” “trouble,” “frustrating,” and “hate” are less frequently used but still notable on the list of negative terms. The word cloud of all positive and negative during the interview process can be found in Multimedia Appendix 2. For sentiment analysis, the median polarity is 0.13 (0.08-0.15), suggesting a slightly positive sentiment overall, and the median subjectivity is 0.51 (0.47-0.53), which indicates a balance between objective and subjective content in the texts (Figure 2).

**Table 1:** The most used positive and negative terms by participants in our pilot study, which explored perceptions of the digital brain health platform, were identified during semistructured interviews conducted virtually through Zoom between April 2022 and July 2023.

Terms			Word count, n		Participants, n
Positive terms					
	Like	540		28	
	Well	207		28	
	Good	152		26	
	Right	136		26	
	Pretty	75		22	
	Better	67		24	
	Fine	67		21	
	Interesting	47		17	
	Easy	45		15	
	Work	43		15	
Negative terms					
	Hard	38		17	
	Problem	37		19	
	Difficult	26		12	
	Inaudible	24		9	
	Bad	24		15	
	Wrong	19		14	
	Uncomfortable	19		11	
	Trouble	18		11	
	Frustrating	18		10	
	Hate	15		7	

## Discussion

### Principal Findings

This study conducted in-depth semistructured interviews with older adults who enrolled in a digital brain health platform and applied NLP to characterize their perspectives on the platform. The findings indicate a positive attitude among participants related to the digital brain health platform.

Older adults stand as pivotal participants in the realm of technology, with their attitudes playing a crucial role in shaping the development and adoption of digital brain health solutions to aid in the treatment and prevention of AD. Previous research delving into the technological attitudes of older adults frequently examines their perspectives on general technologies that do not specifically pertain to cognitive health [16-18], or on their perceptions of a single type of technology [30,31]. However, a growing body of evidence suggests that multimodal data will significantly enhance the predictive capabilities of cognitive-related disorders [3,4]. Thus, the construction of a comprehensive digital phenotyping platform will be important to provide a multidimensional view that could lead to early detection and intervention strategies for delaying or preventing cognitive impairments.

Our study findings of frequent use of terms such as “like,” “well,” and “good” indicate a generally positive reception to the technology and reflect satisfaction or a sense of ease. While the presence of terms such as “difficult” and “hard” are the top negative words that point to some challenges or frustrations as well, the lower frequency of these terms, compared with positive ones, reinforces the overall positive sentiment toward the platform. The sentiment analysis complements these findings by quantitatively measuring the emotional tone and subjectivity in participant responses. The median polarity score of 0.13 (IQR 0.08-0.15), while modestly positive, suggests that on average, participants felt more positively than negatively about the platform. The median subjectivity score indicates a balanced narrative that includes both personal experiences and objective statements about the platforms.

A primary strength of our research lies in surveying older adults after they engaged with a variety of digital technologies over a 2-week period. This extended interaction period allowed participants to form well-rounded opinions. Furthermore, the NLP analysis approach provides a more objective and less time-intensive approach for measuring older adults’ attitudes. NLP’s detailed analysis, extending across word and participant levels, captures the intricacies of sentiment, offering a rich portrayal of users’ emotional responses and overall comfort with the platform.

While our study provides insights into the perspectives of older adults regarding digital health platforms, there were limitations. Selection bias is one concern, as the participants who are more at ease with technology may have been more inclined to engage with the study, potentially leading to an overestimation of the positive sentiment. While our digital brain health platform adopts a participant-driven approach that values users’ preferences in device selection, it may not fully capture the attitudes of a broader spectrum of older adults, particularly those who are less familiar or less comfortable with digital tools—a noted challenge in digital studies [32,33]. This could limit the generalizability of our findings. In future studies, it is crucial to develop strategies that encourage a more diverse group of participants to take part. In addition, some positive words might be used as adverbs or within contexts that alter their intended sentiment. Another notable limitation is the modest sample size of the study, which, combined with the relatively higher education level of the participants, suggests a need for caution in generalizing the findings. While qualitative semistructured interviews typically involve a relatively small sample [34,35], it is important to note that our study design was constrained by the initial readiness of participants in our cohort. This limitation highlights the necessity for a more inclusive approach in subsequent investigations. As a feasibility study, our findings provide a solid foundation for further explorations. Moving forward, it is imperative that future research broadens the enrollment of a more diverse spectrum of participants, such as those with varying educational backgrounds. Expanding the diversity of participants will help ensure that the results are representative of the broader population, thereby enhancing the generalizability and applicability of our findings. In addition, our study is fully data driven and designed to use all available textual data to provide comprehensive, automated insights into participants’ perspectives. Furthermore, the integration of NLP techniques with traditional quantitative research theories represents a promising direction for future research, aiming to extract richer, more nuanced information from interview data.

### Conclusion

This study provides a multifaceted description of older adults’ perspectives on a digital brain health platform. While the overall sentiment is positive, the nuanced data from word counts and sentiment analysis reveal an array of user experiences, underscoring the importance of addressing individual challenges and concerns to enhance the technology’s adoption and efficacy.

## Appendix

Multimedia Appendix 1 STROBE (Strengthening the Reporting of Observational Studies in Epidemiology) checklist.

Multimedia Appendix 2 Positive and negative word clouds of all participants’ interview transcripts were identified during semistructured interviews. Larger and more prominent words indicate a higher frequency of occurrence.

## Abbreviations

AD: Alzheimer disease
BU ADRC: Boston University Alzheimer’s Disease Research Center
HIPAA: Health Insurance Portability and Accountability Act
NLP: natural language processing
STROBE: Strengthening the Reporting of Observational Studies in Epidemiology statement

## Data Availability

The generated datasets for this study could be shared on reasonable request.
