# Supplementary figures and images for: Exploring the Perspectives of Older Adults on a Digital Brain Health Platform Using Natural Language Processing: Cohort Study
Source: JMIR Form Res. 2024 Nov 18;8:e60453. doi: 10.2196/60453 (PMC11612578; doi:10.2196/60453)

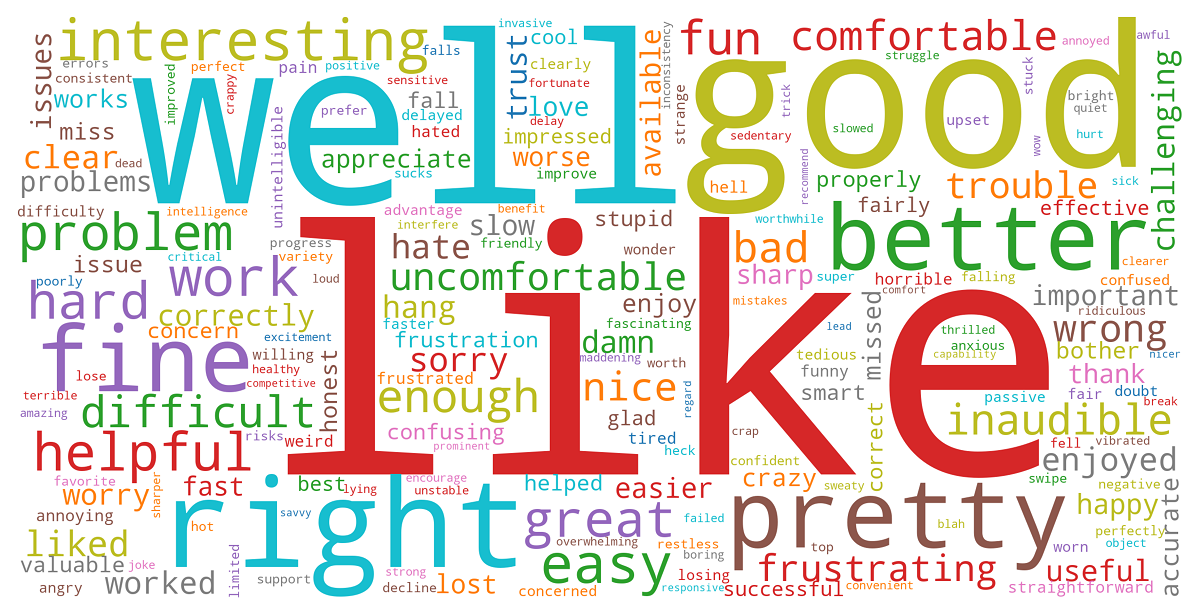

Supplement: Multimedia Appendix 2 [file formative_v8i1e60453_app2.png]
